# PKM2 Interacts With the Cdk1-CyclinB Complex to Facilitate Cell Cycle Progression in Gliomas

**DOI:** 10.3389/fonc.2022.844861

**Published:** 2022-03-22

**Authors:** Shigeo Ohba, Yongjian Tang, Tor-Christian Aase Johannessen, Joydeep Mukherjee

**Affiliations:** ^1^ Department of Neurological Surgery, University of California, San Francisco, San Francisco, CA, United States; ^2^ Department of Neurosurgery, Fujita Health University, Toyoake, Japan; ^3^ Department of Neurosurgery, Xiangya Hospital, Central South University, Changsha, China; ^4^ The Kristian Gerhard Jebsen Brain Tumor Research Centre, Department of Biomedicine, University of Bergen, Bergen, Norway

**Keywords:** PKM2, G2-M arrest, Cdk1, cyclin B, glioma

## Abstract

PKM2 is a phosphotyrosine-binding glycolytic enzyme upregulated in many cancers, including glioma, and contributes to tumor growth by regulating cell cycle progression. We noted, however, that in multiple glioma cell lines, PKM2 knock-down resulted in an accumulation of cells in G2-M phase. Moreover, PKM2 knock-down decreased Cdk1 activity while introducing a constitutively active Cdk1 reversed the effects of PKM2 knock-down on cell cycle progression. The means by which PKM2 increases Cdk1 activity have not been described. Transient interaction of T14/Y15-phosphorylated Cdk1 with cyclin B allows Cdk7-mediated pT161 Cdk1 phosphorylation followed by cdc25C-mediated removal of pT14/Y15 and activation of Cdk1 in cycling cells. In the present course of investigation, PKM2 modulation did not influence Cdk7 activity, but phosphotyrosine binding forms of PKM2 co-immunoprecipitated with pY15-containing Cdk1-cyclinB and enhanced formation of active pT161 Cdk1-cyclin B complexes. Moreover, exogenous expression of phosphotyrosine binding forms of PKM2 reversed the effects of PKM2 knock-down on G2-M arrest. We here show that PKM2 binds and stabilize otherwise transient pY15-containing Cdk1-cyclinB complexes that in turn facilitate Cdk1-cyclin B activation and entry of cells into mitosis. These results, therefore, establish metabolic enzyme PKM2 as a direct interactor and activator of Cdk1-cyclin B complex and thereby directly controls mitotic progression and the growth of brain tumor cells.

## Introduction

Conversion of phosphoenolpyruvate (PEP) to pyruvate the final rate-limiting step of glycolysis is catalyzed by Pyruvate kinase (PK) (1), a metabolic enzyme that exists as homotetrameric M1 form (PKM1) expressed in normal differentiated tissues, including the brain with a high affinity for PEP and generate high levels of pyruvate (2–4). Whereas proliferating cells from tumors as well as fetal tissues expressed M2 isoform (PKM2) (5, 6) that oscillates between a glycolytically active tetramer and a more loosely associated, glycolytically-inactive, monomeric/dimeric form (7). In tumor cells, PKM2 predominantly exist in monomeric/dimeric forms due to low levels of allosteric activator fructose bisphosphate (FBS) and due to the presence of phosphorylated PKM2 at Y105 (8) that have an increased binding affinity for pY-containing proteins (9). The resultant decrease in PK activity spares the intermediates of glycolysis for biosynthetic processes and helps create a metabolic environment suitable for tumor cell proliferation.

In addition to altering metabolism, the conversion of tetrameric PKM2 to dimeric PKM2 reveals a variety of non-metabolic PKM2 functions that contribute to tumor cell growth. Dimeric PKM2, unlike PKM1 or tetrameric PKM2, has protein kinase activity and uses PEP as a donor to phosphorylate histone H3 and Stat3 (10, 11). This results in the increased transcription of a variety of genes encoding proteins such as cyclin D1 and c-myc that promote cell cycle entry. Dimeric PKM2 is also a pY-binding protein and uses a domain that includes PKM2 K433 to bind to pY133 β-catenin and pY200 HuR (11–13). Furthermore, mutant forms of PKM2 that have enhanced protein kinase activity stimulate cell growth. At the same time, those defective in pY-binding fail to rescue tumor cells from the growth inhibition caused by PKM2 suppression (9, 10, 12). These results suggest that the non-metabolic effects of PKM2, particularly transcription reprogramming, co-operate with PKM2-induced metabolic changes to facilitate cell cycle entry and tumor cell growth.

Consistent with a role for PKM2 in tumor growth, several groups have noted that suppression of PKM2 levels leads to decreased tumor cell proliferation (9, 11, 14). We noted, however, that tumor cells expressing shRNA constructs targeting PKM2 do not have generalized growth defects but rather have very specific cell cycle defects, accumulating with a greater than 2N DNA content and in a manner consistent with an inability to enter mitosis (13, 15). Entry into mitosis is an extensively studied yet incompletely understood process that represents the last barrier to proliferation, particularly in tumor cells that frequently lack a G1 checkpoint (16). Furthermore, because entry into mitosis requires a single cyclin-dependent kinase (Cdk1) whose activity is extensively regulated by post-transcriptional events, we considered the possibility that PKM2 uses a non-metabolic function other than transcriptional regulation to control entry into mitosis and to promote tumor cell growth. In this report, we show that PKM2 is an integral component of the Cdk1-cyclin B complex that controls mitotic entry, binding independently of its protein kinase or metabolic activity to nuclear Y15-phosphorylated Cdk1-cyclin B complexes to facilitate Cdk1 activation. The increased Cdk1 activation, in turn, facilitates the progression of tumor cells into mitosis. These findings, therefore, place PKM2 in direct contact with the cell cycle machinery and identify a novel means by which PKM2 links metabolism to the control of the cell cycle and tumor cell growth.

## Materials and Methods

### Cell Culture

U87MG, T98G, LN319, GBM6, GBM39 human glioma cells were provided by the UCSF Brain Tumor Center Tissue Core and were cultured as described (13, 15). The generation of NHAs expressing E6/E7/hTERT plus H-RasV12 (Ras astrocytes) has been described previously (17, 18).

### Modulation of PKM2 and Cdk1 Expression

Non-targeted or one of five different lentiviral PKM2 shRNA were used to infect U87, T98G, and LN319 cells as described previously (13, 15). Following puromycin selection (1 μg/ml, two weeks) and expansion, the 2 clonal populations exhibiting the lowest PKM2 expression relative to controls for each cell type were chosen for further study. The parental and PKM2 knock-down cells were also transiently transfected (Fugene 6) with one of four different forms of GFP-Cdk1 (Cdk1-AY, Cdk1-TF, Cdk1-AF, and Cdk1-WT), or one of four different forms of mouse PKM2 (PKM2, K399E, K367M, R399E). Cell cycle analysis and lysate preparation was performed 48 hours after transfection.

### Cell Synchronization and Cell Cycle Analysis

Cells were synchronized by incubation for 48 hrs in serum-free DMEM-H21 media followed by a release in 10% FBS-containing media and collection at the indicated time points. In some experiments, cells were also incubated with DASA-58 (40 μM, 3 hrs, Millipore) prior to harvest. Mitotic cells were collected from the media after a 2-3 min shake of the flasks 24 hrs after serum-stimulation, with a greater than 90% purity of the population. For mitotic count, cells were fixed and stained with phospho (Ser 28) histone H3.3 antibody (Cell Signaling) and analyzed by flow cytometry. Cell cycle distribution was assessed in fixed, propidium iodide-labeled cells subjected to flow cytometry using a FACSCalibur (BD Biosciences) in combination with Flowjo software (Treestar) (15).

### Protein Extraction, Cross-Linking, Immunoprecipitaton, and Western Blot Analysis

Protein lysates from control or DASA-58 (0-100 uM, 3 hrs) treated cells were prepared in lysis buffer (50mM HEPES, pH7.0, 150mM NaCl, 10% Glycerol, 1% Triton-X, 1mM EDTA, 100mM NAF, 10mM NaPPi) supplemented with protease and phosphatase inhibitors (Roche). For cross-linking studies, cell lysates (100μg) were incubated with freshly prepared glutaraldehyde (0.125% final concentration, 5 minutes, 37°C). Following termination of the reaction, the cross-linked proteins were solubilized by the addition of an equal volume of Laemmli sample buffer. For immunoprecipitation, protein lysates were pre-cleared with protein A/G-agarose beads (Santa Cruz, 3 h, 4°C), then incubated with primary antibody (16 h, 4°C). The immune complexes were precipitated for 2 h at 4°C with protein A/G-agarose beads. In control samples, the primary antibody was substituted with control IgG (rabbit or mouse depending on the source of the primary antibodies). Immunoprecipitates were washed four times with RIPA buffer containing 0.5 M NaCl and 2% SDS and three times with PBS and then resuspended in Laemmli buffer. Proteins were separated on 4-20% Tris-Glycine gradient polyacrylamide gels (Invitrogen) and transferred onto Immuno-Blot PVDF membranes (Bio-Rad Laboratories). Membranes were then incubated in blocking buffer (1X TBS containing 5% milk and 0.05% Tween-20, 2 hours), probed overnight with antibodies specific for PKM2 (1:1000), β-actin (1:20,000), cdc25C, phospho (S216) cdc25C, cyclin B1 (all Cell Signaling), GFP (ThermoFisher), or Cdk1(Santa Cruz), washed, then incubated with appropriate horseradish peroxidase-conjugated secondary antibodies (Santa Cruz Biotechnology). Antibody binding was detected by incubation with ECL reagents (Amersham Pharmacia Biotech).

### Cdk1-Cyclin B, cdk7, and PK Activity Assays

For Cdk1-cyclin B activity analysis, cell lysates were diluted and pipetted into recombinant Cdc7 pre-coated wells (MBL International Corp), after which Mg2+ and ATP were added, and the amount of phosphorylated substrate was measured by binding to an anti-phospho-Cdk7 (T 376) antibody and a horseradish peroxidase-conjugated anti-mouse IgG. The catalyzed color reaction was quantified by spectrophotometry and used to determine the relative amount of Cdk1-cyclinB activity in the samples following the manufacturer’s directions (MBL International Corp). The Cdk7 activity was measured by using recombinant Cdk7/Cyclin H/MAT1 enzyme, Cdk substrate peptide, and luminescence was detected using the microplate reader as per the manufacturer (BPS Bioscience, San Diego) instruction. PK activity was measured as previously described (13).

### Statistical Analysis

*In-vitro* experiments were performed in triplicates. Means and standard errors were computed. The unpaired Student’s t-test was applied for comparing two groups while a one-way ANOVA test with *post hoc* Turkey-Kramer multiple comparisons test was used to evaluate multiple groups. *P*<0.05 was considered statistically significant.

## Results

### Loss of PKM2 limits Cdk1-CyclinB Activation and G2-M Cell Cycle Arrest

**Figure 1:**
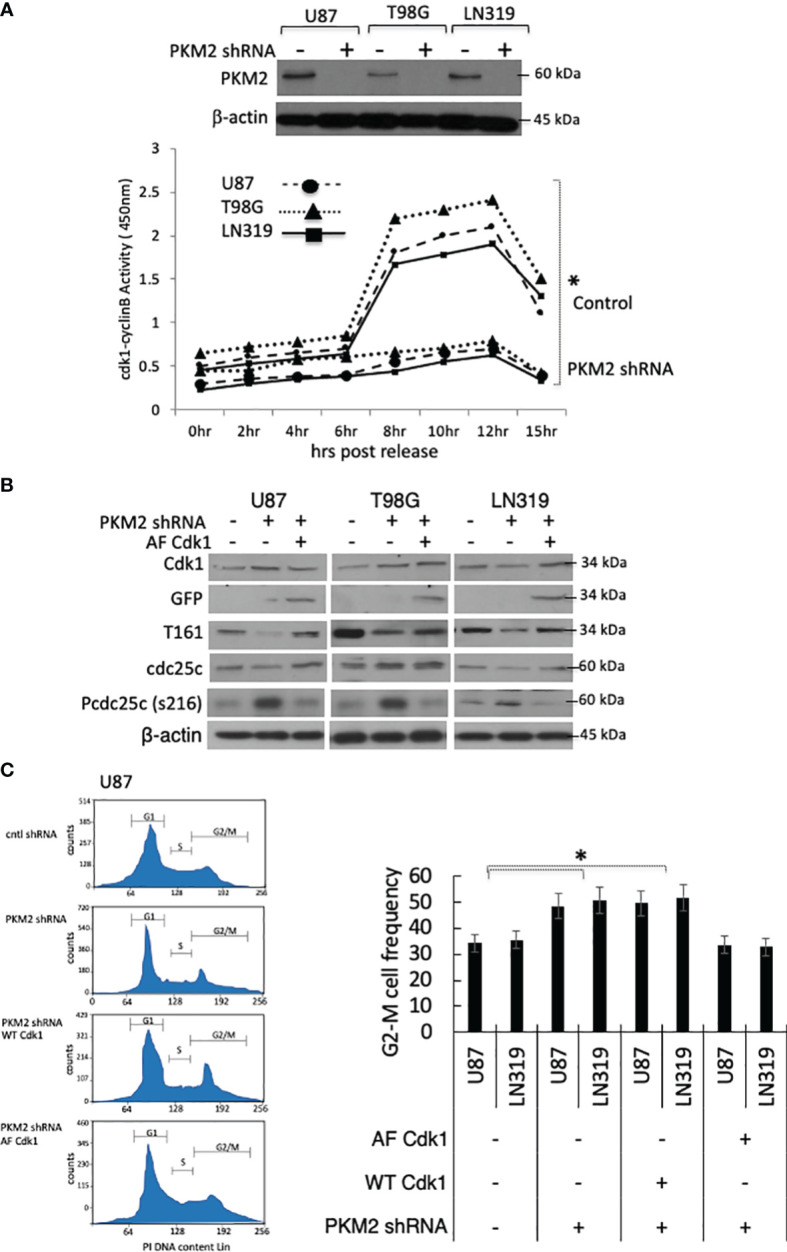
Loss of PKM2 limits Cdk1-cyclinB activation and G2-M cell cycle arrest. **(A)** U87, T98, or LN319 glioma cells were lentivirally infected with a scrambled shRNA (-) or constructs encoding shRNAs targeting human PKM2 (+). Following drug selection, polyclonal populations were examined by Western blot for PKM2 and β-actin expression. Cdk1-cyclin B activity in lysates from synchronized, serum-stimulated control and PKM2 knock-down U87, T98G and LN319 cells as determined by an *in vitro* kinase assay. **(B)** Levels of Cdk1, GFP, pT161 Cdk1, Cdc25C, pS216 Cdc25C and β-actin in total cell lysates from unsynchronized cells expressing scrambled shRNA, or PKM2 shRNA and subsequently manipulated to express a blank or AF Cdk1-encoding construct. **(C)** The distribution of cells in various phases of the cell cycle was determined by FACS-based analysis on U87 and LN319 cells expressing scrambled shRNA or PKM2 shRNA, and expressing WT or constitutively active (AF) Cdk1. Representative cell cycle plot of all treatment groups of U87 cells in left panel and G2-M values from all treatment group of U87 and LN319 cell lines are presented on the right panel. Values are presented as the mean ± standard error of mean of three determinations. PI, propidium iodide. *p<.05, n=3.

PKM2 knock-down cells accumulated in the G2-M phase of the cell cycle, and Cdk1 is the only cyclin-dependent kinase required for entry into mitosis (19, 20). We, therefore, considered the possibility that PKM2 uses the regulation of Cdk1 for mitotic progression. To address this possibility, we did a stable knock-down of PKM2 using lentiviral shRNA in U87, T98G, and LN319 glioma cell lines. As shown in the Western blot in **Figure 1A** top panel, the lentiviral introduction of shRNA targeting PKM2 (+) resulted in significant decreases in PKM2 expression relative to scramble (-) controls in each cell lines. We next measured Cdk1 activity in lysates of control and PKM2 knock-down cells after release from serum starvation-induced synchronization. As cells approach mitosis, levels of cyclin B rise and the Cdk1 transiently associated with cyclin B becomes activated by phosphorylation on Thr161 by Cdk7 (21, 22). This activation is opposed by Myt1- and Wee1-mediated inhibitory phosphorylations of Cdk1 at T14 and Y15 (23), respectively, which can be removed by the cdc25 family of phosphatases, primarily cdc25C (24). Control cells showed the expected rise in Cdk1-cyclin B activity associated with entry into mitosis 8-10 hrs after addition of serum (**Figure 1A**). This pulse of Cdk1 activity was significantly reduced in PKM2 knock-down cells, suggesting a defect in Cdk1 activation in these cells. Consistent with this observation, PKM2 knock-down cells also contained significantly less Cdk1 activated by T-loop (thr161) phosphorylation (**Figure 1B**). PKM2 knock-down cells also retained more cdc25C in a phosphorylated (ser216) form that is a substrate for 14-3-3-dependent nuclear export and cannot contribute to the removal of the inactivating phosphorylations of Cdk1 at T14 and Y15 (**Figure 1B**) (25). Furthermore, increasing Cdk1 activity by the introduction of a constitutively active GFP-tagged Cdk1 mutated to eliminate the possibility of inactivating phosphorylations at T14 and Y15 (AF Cdk1) (26) reversed the increases in pcdc25C levels caused by PKM2 knock-down (**Figure 1B**). Representative flow plot of U87 glioma cells in which PKM2 levels were suppressed (PKM2 shRNA) exhibited an accumulation of G2-M cells based on their cell cycle analysis relative to scramble controls (-). There is a slight decrease in the percentage of cells in the G0/G1 and S phase in the PKM2 knock-down group compared to the control group. Introducing wild type Cdk1 (WT Cdk1) was unable to rescue the G2-M arrest in PKM2 knock-down U87 cells, but mutated AF-Cdk1 that are constitutively active was able to reverse the effects of PKM2 loss on G2-M arrest along with the little changes in G0/G1 and S phase (**Figure 1C**, left panel). Quantitative analysis of cells in G2-M phase also demonstrated a significant increase in PKM2 knockdown group which was rescued by AF-Cdk1 but not with WT Cdk1 in both U87 and LN319 cell lines (**Figure 1C**, right panel). These results show that Cdk1 activity is stimulated by PKM2 and that Cdk1 is a key means by which PKM2 regulates cell cycle progression.

### PKM2 Physically Interacts With the Cdk1-CyclinB Complex

**Figure 2:**
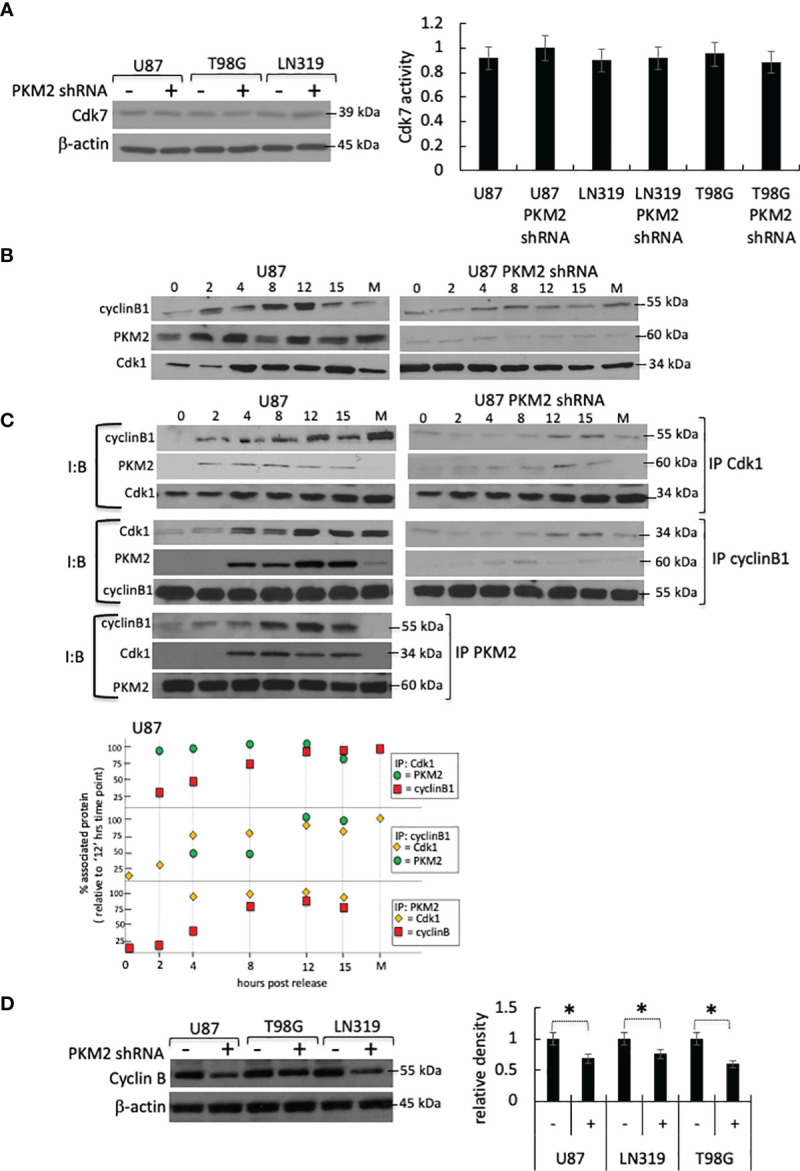
PKM2 physically interacts with the Cdk1-cyclin B complex. **(A)** Levels (top) and activity (bottom) of Cdk7 in control and PKM2 knock-down U87, T98G and LN319 cells 10 hrs following release from serum deprivation-induced arrest. **(B)** Levels of cyclin B1, PKM2, and Cdk1 in total cell lysates in control or PKM2 shRNA U87 cells measured at the times indicated following release from serum deprivation-induced arrest. **(C)** Levels of Cdk1, cyclin B, or PKM2 from Cdk1, cyclin B or PKM2 immunoprecipitates measured at the times indicated following release of control or PKM2 shRNA U87 cells from serum deprivation-induced arrest. Data in M lanes were derived from cells isolated by mitotic shake-off. I:B = Immuno Blot, IP = immunoprecipitate. Bottom panel: Densitometric analysis of Cdk1, cyclin B, or PKM2 levels in U87 cells from Cdk1, cyclin B or PKM2 immunoprecipitates. **(D)** Western blot analysis of cyclin B1 in control and PKM2 knock-down U87, T98G and LN319 cells. Densitometric analysis (bottom right panel) of western blot demonstrating levels of cyclin B1 in different experimental groups. *p<.05, n=3.

PKM2 could increase Cdk1 T161 phosphorylation and activation indirectly by increasing the activity of the Cdk1-activating Cdk7 kinase. The levels and activity of Cdk7, however, were comparable in both control and PKM2 knock-down cells (**Figure 2A**). Alternatively, PKM2 could increase Cdk1 activity by increasing the levels of Cdk1 or cyclin B, or by stimulating the formation or activation of the Cdk1-cyclin B complex. To address these possibilities, control, and PKM2 knock-down cells were serum-starved, and lysates were collected 0-15 hrs after release into the complete serum. In the total cell lysates of control cells, levels of cyclin B rose as the cells neared entry into mitosis around 10 hrs post-release (**Figure 2B**, left panel). In control lysates in which total Cdk1 was immunoprecipitated, then assessed by Western blot with a cyclin B antibody, an interaction between Cdk1 and cyclin B was observed in between 2-15 hrs post-release (**Figure 2C**, top left panels and densitometric analysis at the bottom panel) and in a time frame consistent with the increase in Cdk1 activity in these cells (**Figure 1A**). This same interaction was apparent in the reverse experiments in which cyclin B was immunoprecipitated followed by Western blot analysis using a Cdk1 antibody (**Figure 2C**, left middle panels and densitometric analysis at the bottom panel). In patient-derived xenograft (PDX) cell line GBM6 and GBM39, from Cdk1immunoprecipitate and cyclin B co-immunoprecipitate, the interaction between Cdk1, cyclin B, and PKM2 in a similar time frame was confirmed as like control U87 cell line (**Supplementary Figure 1A**). Normal human astrocytes (NHAs) that slowly proliferate for a very limited passage number express mostly PKM1 and a very little PKM2 and can serve as a negative control (**Supplementary Figure 1B**). The generation of genetically modified NHAs that express E6-E7-hTERT plus H-RasV12 and are transformed (17, 18) mainly express PKM2, and a very little PKM1 will be a positive control cell line for Cdk1, cyclin B, and PKM2 interaction (**Supplementary Figure 1B**). IP-Co-IP experiments using lysates of genetically modified NHA-e6-e7-hTERT-Ras cells demonstrated strong interaction between cdk1, cyclin B, and PKM2 between 2-15 hrs post-release (**Supplementary Figure 1C**, right panel). In PKM2 knock-down U87 cells, levels of total Cdk1 were comparable to those in control cells, while levels of cyclin B were approximately 30% lower (**Figures 2B, D**). Minimal cyclin B, however, was found in Cdk1 immunoprecipitates, and very little Cdk1 was found in cyclin B immunoprecipitates, despite the presence of both proteins in the lysates (**Figure 2C**, right panels). Similarly, in immunoprecipitates of Cdk1 and cyclin B from unmodified NHAs that express very little PKM2, minimal cyclin B or Cdk1 were found, respectively demonstrating little or no interactions (**Supplementary Figure 1C** left panel). The inability of Cdk1-cyclin B complexes to form in the absence of PKM2 suggested that PKM2 might directly interact with one or both of these molecules to facilitate the complex formation, activation, and cell cycle progression. Consistent with this idea, PKM2 was found in both Cdk1 and cyclin B immunoprecipitates in U87 cells (**Figure 2C**, left panels), PDX cell lines (GBM6 and GBM39) (**Supplementary Figure 1A**), and positive control NHA-e6-e7-hTERT-Ras cells ((**Supplementary Figure 1C**, right panel), and conversely, cyclin B and Cdk1 were found in PKM2 immunoprecipitates (**Figure 2C**, bottom left panel). PKM2, however, was not associated with either cyclin B or Cdk1 until these proteins became associated with one another as the cells began to approach mitosis and was not associated with either protein in immunoprecipitates from cells undergoing mitosis (M, **Figures 2B, C**, left panels). These results show that PKM2 physically interacts with a component of the Cdk1-cyclin B complex and that this interaction facilities complex formation/activation and entry into mitosis.

### Pharmacologic Activation of PKM2 Suppresses Cdk1-CyclinB Complex Levels

**Figure 3:**
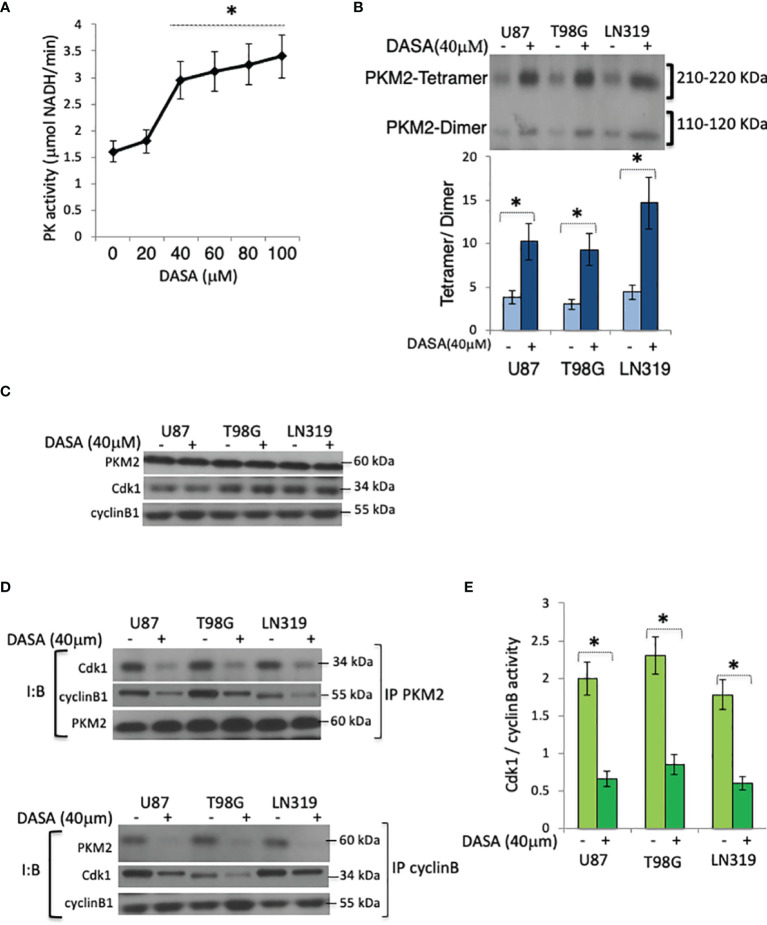
Pharmacologic activation of PKM2 suppresses Cdk1-cyclin B complex levels. **(A)** PK activity in U87 cells exposed to DASA-58 (0-100 μM, 3 hrs) as measured by an *in vitro* PK assay. **(B)** Levels of tetrameric and dimeric PKM2 in chemically cross-linked lysates of U87, T98G, or LN319 cells10 hrs following release from serum deprivation-induced arrest and incubated with vehicle (DMSO) (-) or DASA-58 (40 μM) 3hrs before harvest. The lower panel is the densitometric analysis of the data in the upper panel displayed as the tetramer/dimer ratio, **(C)** Levels of PKM2, Cdk1, and cyclin B in vehicle(DMSO)(-) or DASA-58-treated (40 μM, 3 hrs) U87, T98G, or LN319 cells from panel **(B) (D)** Levels of Cdk1, cyclin B1 and PKM2, in whole cell PKM2 (top) or cyclin B (bottom) immunoprecipitates from cells 10 hrs after release from serum deprivation-induced arrest and which were exposed to vehicle (DMSO) (-) or DASA-treated (40 μM) for the last three hrs of the study. **(E)** Cdk1-cyclin B activity in lysates from panel **(B)** I:B, Immuno Blot; IP, immunoprecipitate. *p <.05, n=3.

Growth signaling and the subsequent metabolic needs of tumor cells drive the conversion of PKM2 from a glycolytically active tetramer to a glycolytically inactive dimer (10). To determine if this conversion plays a role in Cdk1-cyclin B complex formation, tumor cells were incubated with N,N’-diarylsulfonamide NCGC00185916 (DASA-58), a PKM2 activator that binds PKM2 subunits and promotes tetramer formation (7), after which the cells were monitored for effect on the Cdk1-cyclin B complex levels. PK activity was enhanced by exposures of DASA-58 (40 μM, 3hrs) (**Figure 3A**) that also significantly increased the tetrameric: dimeric PKM2 ratio (**Figure 3B**). While the exposure of cells to DASA-58 had no effect on total amounts of Cdk1, cyclin B or PKM2 in serum-starved cells 10 hrs after serum addition (**Figure 3C**), it significantly decreased the amount of cyclin B and Cdk1 that could be immunoprecipitated with PKM2 (**Figure 3D**, top panel), decreased the amount of PKM2 and Cdk1 that could be immunoprecipitated with cyclin B (**Figure 3D**, bottom panel), and decreased Cdk1-cyclin B activity in the PKM2 immunoprecipitates (**Figure 3E**). These results show that the shift from tetrameric to dimeric PKM2 facilitates Cdk1-cyclin B complex formation, and that these events can be inhibited by the pharmacologic activator of PKM2.

### A Y15 Cdk1-PKM2 Interaction Facilitates Cdk1-CyclinB Activation and Cell Cycle Progression

**Figure 4:**
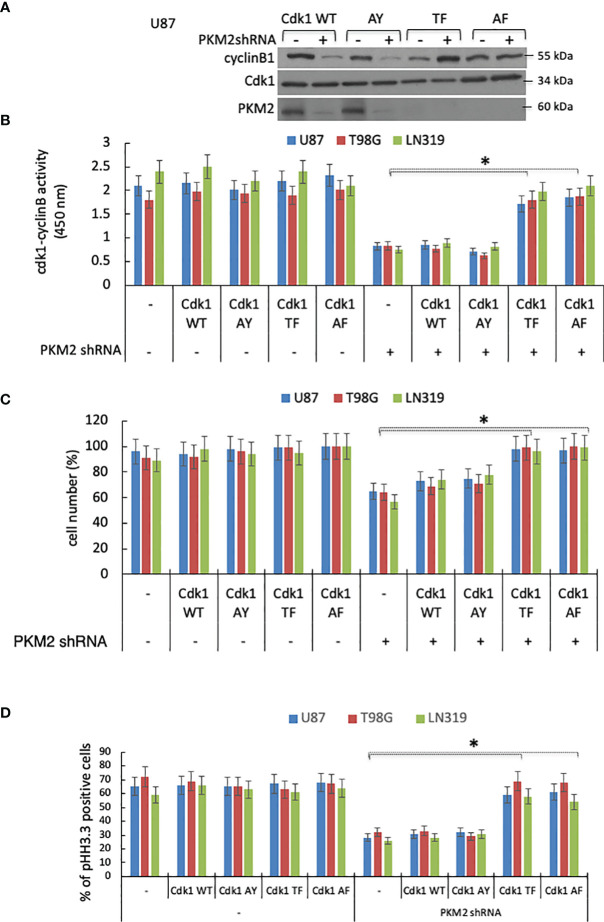
A Y15 Cdk1/PKM2 interaction facilitates Cdk1-cyclin B activation and cell cycle progression. **(A)** Levels of cyclin B Cdk1, and PKM2 in nuclear GFP immunoprecipitates from control or PKM2 shRNA-expressing U87 cells expressing GFP-tagged WT, AY, TF or AF forms of Cdk1 measured 10 hrs after release of cells from serum deprivation-induced cell arrest. **(B)** Cdk1-cyclin B activity in lysates from U87, T98G and LN319 cells expressing GFP-tagged WT, AY, TF or AF forms of Cdk1 measured 10 hrs after release of cells from serum deprivation-induced cell arrest as determined by an *in vitro* kinase activity assay. **(C)** Normalized cell number in control and PKM2 shRNA-expressing U87, T98G and LN319 cells expressing GFP-tagged WT, AY, AF, or TF forms of Cdk1 48 hrs after plating. **(D)** cells from panel-b were assessed for the percentage of mitotic pHH3.3+ cells by FACS. *p <.05, n=3.

PKM2 is a pY-binding protein, and although cyclin B has not been reported to contain pY residues, Cdk1 is phosphorylated on Y15 by Wee1 (27, 28). We therefore considered the possibility that PKM2 may drive cell cycle progression by binding Y15 phospho-Cdk1 in Cdk1-cyclin B nuclear complexes and facilitating their activation. To address this possibility, PKM2 WT and PKM2 knock-down cells were infected with constructs encoding GFP-tagged wild-type Cdk1 or forms of Cdk1 mutated to prevent inactivating phosphorylation at T14 (AY), Y15 (TF) or both (AF) (27). The cells were then synchronized by serum starvation, returned to serum-containing media, and harvested at a time (10 hrs post serum addition) at which PKM2-Cdk1-cyclin B complexes had formed in control cells. Nuclear immunoprecipitated GFP-Cdk1 complexes from control cells expressing the GFP-tagged wild-type or AY Cdk1 contained PKM2 while those from cells expressing GFP-tagged Cdk1 proteins mutated to prevent phosphorylation at Y15 (TF or AF) did not (**Figure 4A**). Furthermore, although the immunoprecipitated Cdk1-cyclin B complexes from all groups displayed Cdk1/cyclin B activity when PKM2 was present, only those complexes which could not be inactivated by Y15 phosphorylation (TF, AF) retained activity in the absence of PKM2 (**Figure 4B**), and only the TF and AF forms of Cdk1 could rescue cells from PKM2 shRNA-induced growth suppression (**Figure 4C**). Most of the PKM2-knockdown cells were unable to progress into mitosis (phospho-histone H3.3+ cells) 12 hrs. following release compared to control cells (**Figure 4D**), and only the TF and AF forms of Cdk1 could rescue cells to reenter mitosis from PKM2 shRNA-induced G2-M arrest. The binding of PKM2 to the Cdk1-cyclin B complex therefore is dependent on the presence of Cdk1 that can be phosphorylated at Y15, and this PKM2-pY15Cdk1-cyclin B interaction appears critical for subsequent Cdk1 activation.

### PKM2 Uses Its pY-Binding Ability to Facilitate Cdk1-CyclinB Activation and Cell Cycle Progression

**Figure 5:**
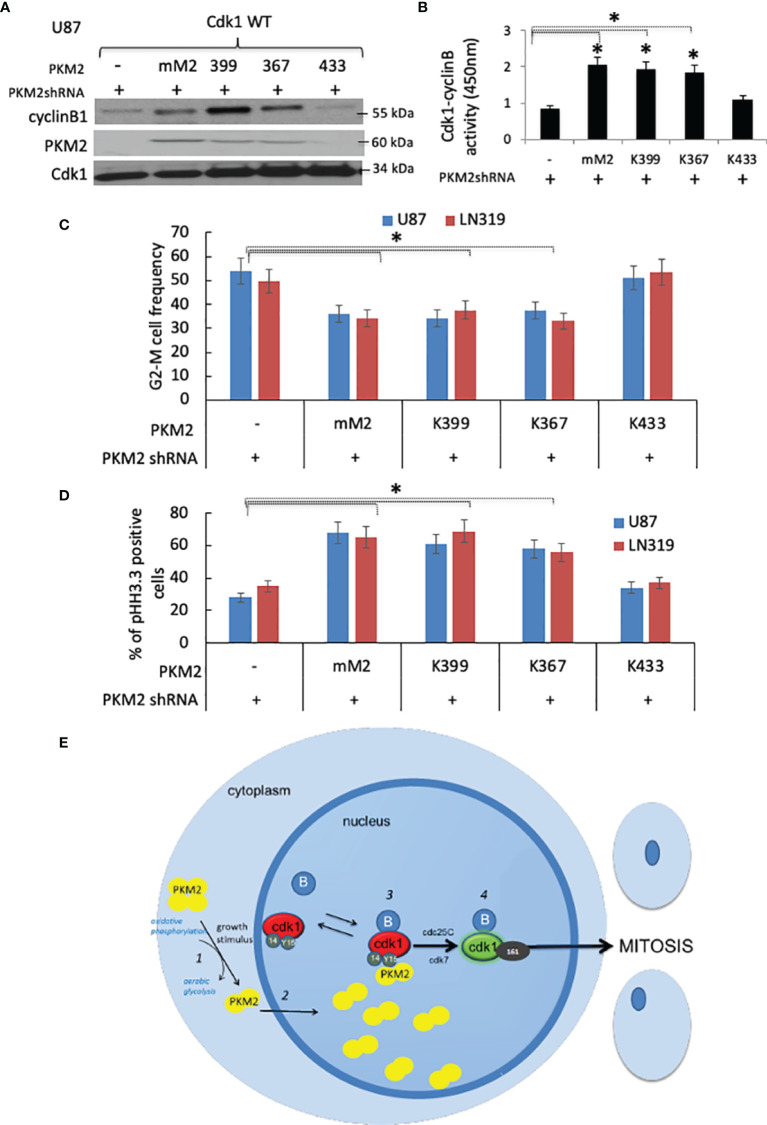
PKM2 uses its pY-binding ability to facilitate Cdk1-cyclin B activation and cell cycle progression **(A)** Levels of cyclin B1, PKM2, Cdk1, and **(B)** Cdk1-cyclin B activity in nuclear GFP immunoprecipitates from PKM2 shRNA-expressing U87 cells expressing GFP-tagged WT Cdk1 and mouse WT (mM2) or R399E, K367M, or K433E forms of PKM2 measured 10 hrs after release of cells from serum deprivation-induced arrest. **(C)** FACS-based cell cycle distribution in U87 and LN319 cells containing constructs encoding a scrambled shRNA or PKM2 shRNA, mouse PKM2, or various mutant forms of PKM2. *p <.05, n=3. **(D)** cells from panel-C were assessed for the percentage of mitotic pHH3.3+ cells by FACS **(E)** Proposed model by which PKM2 interacts with the Cdk1-cyclin B complex to drive mitotic progression in tumor cells. Extracellular events that stimulate tumor cell growth trigger dimerization of PKM2 and a shift in metabolism (1). PKM2 dimers move into the nucleus (2) where they interact with Y15-phosphorylated Cdk1-cyclin B complexes (3). The interaction, perhaps through complex stabilization increases the likelihood of Cdk1 activation (4). Cdk1 activation allows the cellular metabolic state to match the cellular proliferative state.

To better understand which PKM2 activities are critical for Y15 Cdk1 binding, PKM2 knock-down cells were infected with constructs encoding GFP-tagged wild-type Cdk1 and either shRNA-resistant murine WT PKM2 or forms of PKM2 with altered pY binding or kinase activity; K433E, which lacks pY binding but retains both protein kinase and pyruvate kinase activities (12), R399E, which binds pY but because of reduced ability to form tetramers has enhanced protein kinase activity and reduced pyruvate kinase activity (10), or K367M, which retains pY binding in the absence of protein kinase and pyruvate kinase activities (9). The cells were then synchronized by serum starvation, returned to serum-containing media, and harvested at 10 hrs post serum addition, after which nuclear GFP (Cdk1) immunoprecipitates were analyzed for the presence of GFP-Cdk1, PKM2, cyclin B1, and Cdk1-cyclinB activity. Exogenous expression of WT murine PKM2 (mM2) in cells in which endogenous PKM2 expression was suppressed resulted in high levels of GFP-immunoprecipitable Cdk1-cyclin B-PKM2 complexes (**Figure 5A**) and Cdk1-cyclin B activity relative to cells lacking exogenous PKM2 expression (**Figure 5B**). Exogenous expression of kinase-dead (K367) and protein kinase-enhanced (R399) forms of PKM2 that, like WT PKM2 retained pY binding also enhanced Cdk1-cyclin B-PKM2 complex formation and Cdk1-cyclin B activity, while expression of the glycolytically active K433, which cannot bind pY, failed to do so (**Figure 5B**). Consistent with this data, only the forms of PKM2 capable of binding pY (mM2, K367, R399) could, independently of their metabolic and protein kinase activities, reverse the accumulation of cells in G2-M along with a slight increase in G0/G1 and S phase (**Figure 5C**), caused by PKM2 depletion. Significantly less mitotic (phospho-histone H3.3+) cells in PKM2 knock-down group were reversed by overexpressing WT murine (mM2), kinase-dead (K367), and protein kinase-enhanced (R399) forms but not with the form of PKM2 that were unable to bind pY (**Figure 5D**). Collectively these results show that dimeric PKM2 uses its pY-binding ability to increase Cdk1-cyclin B activation to facilitate tumor cell entry into mitosis (**Figure 5E**).

## Discussion

Tumor cell growth requires co-ordination between the factors that control metabolism and the factors that control cell cycle progression. PKM2 is a key regulator of the cellular metabolic state, and in response to growth factor signaling is converted from a tetrameric pyruvate kinase to a dimeric/monomeric protein kinase (10). This conversion conserves the intermediates of glycolysis for biosynthesis, but also allows the protein kinase activity of PKM2 to phosphorylate proteins that indirectly regulate the transcription of growth-related genes (11). The results presented here show that PKM2 acts more directly and centrally on the cell cycle machinery than previously suspected, binding independently of its kinase activity to Y15-phosphorylated Cdk1 in nuclear Cdk1-Cyclin B complexes to facilitate complex activation and cell cycle progression. These studies therefore directly link metabolic regulation to cell cycle machinery and tumor cell growth.

In addition to revealing new PKM2 targets and functions, the present work also identifies PKM2 as an integral component of the Cdk1-cyclin B complex that controls progression into mitosis. PKM2, preferentially in its dimeric form, appears in complexes with Cdk1 and cyclin B in a manner dependent on the presence of a phosphorylatable Y15 Cdk1, and the ability of PKM2 to bind pY-containing peptides. PKM2-containing complexes appear as Cdk1 and cyclin B assemble and become activated in the nucleus approaching entry into mitosis, but are not found in mitotic cells, presumably because in mitosis all Cdk1-cyclin B complexes are activated and pY15 Cdk1 levels are minimal (29). PKM2 also does not associate with Cdk1 at early time points following serum stimulation although it is unclear if this is because of a lack pY15Cdk1-cyclin B complexes or a lack of nuclear pY15Cdk1 available for binding. Alternatively, the generation of Y15Cdk1, the formation of Cdk1-cyclin B complexes, and the binding of PKM2 may occur simultaneously, although more detailed approaches would be required to address this point. While these studies are the first to show that a regulator of cellular metabolism is also an intimate part of the Cdk1-cyclinB complex, the findings are consistent with studies in which PKM2 and cyclin B were identified as binding partners of Cdk1 in post-mitotic *Xenopus laevis* oocytes, but not in the same oocytes in M phase (30).

If PKM2 is an integral component of Cdk1-cyclin B complex, how does its binding bring about an increase in Cdk1 activity and changes in cell cycle progression? With the caveat that Cdk1 activation is an incompletely understood process, the present results could be explained in three different but not mutually exclusive ways. First, PKM2 binding to the Cdk1-cyclin B complex could influence the activities of the enzymes (Cdc25C, Cdk7, Wee1, Myt1) that interact with the complex and lead to Cdk1 activation. In the present studies, however, PKM2 suppression had no effect on Cdk7 activity. Furthermore, although Cdk1 inactivates Wee1 and causes nuclear retention of Cdc25C (25, 30), these actions are a consequence of Cdk1 activation, and not likely an explanation for how PKM2 initiates increases in Cdk1 activity. Second, PKM2 binding to the Cdk1-cyclin B complex may favor nuclear accumulation and subsequent activation of the complex. Y15 phosphorylated Cdk1-cyclin B complexes are in a state of constant flux between the cytoplasm and the nucleus (31), and binding of dimeric PKM2 in the nucleus could limit export and favor Cdk1 activation. Nuclear Cdk1-cyclin B complex levels, however, are regulated primarily by nuclear import rather than nuclear export (31), and as such it is unclear how primarily nuclear forms of PKM2 could regulate the process, although this possibility cannot formally be excluded. Finally, PKM2 may stabilize nuclear pY15Cdk1-cyclin B complexes, making them more likely to be acted upon by Cdk7 and/or Cdc25C. Cdk1 is T161 phosphorylated in the nucleus by Cdk7 simultaneously with its binding to cyclin B, and the processes are mutually dependent (32). Most nuclear Cdk1-cyclin B complexes in G2, however, are phosphorylated at all three regulatory sites (14, 15, and 161), suggesting that the removal of the T14 and Y15 inhibitory phosphorylations from Cdk1 represents the final activating event (33). The observation that pT14 is removed before pY15 (34), coupled with the finding that PKM2 is needed to activate only those forms of Cdk1 that can be phosphorylated at Y15, suggests that PKM2 binding and stabilization of the Cdk1-cyclin B complex may allow Cdc25C more time for the specific removal of pY15. It will be of interest to examine this process in more detail, and to determine how it is accomplished in the absence of PKM2 kinase activity.

In addition to adding to our understanding of the links between metabolism, cell cycle regulation, and tumor cell growth, the results and the model presented suggest new areas of investigation. First, given the importance of the PKM2 pY binding function, and the ability of DASA-58 to reduce Cdk1-cyclin B complex formation, it will now be important to determine how pharmacologic activation of PKM2 alters PKM2 localization and pY binding. Similarly, although DASA-58 decreases Cdk1-cyclin B complex formation in tumor cells, it does not under normoxic conditions alter cell growth (7). It therefore appears likely that additional undefined factors contribute to the regulation of the Cdk1-cyclin B complex and the setting of the cellular proliferative state. Although this present work defined PKM2 as an integral component of the fundamental cell cycle regulatory complex in gliomas, PKM2 is over-expressed in other types of cancers, and most likely as like gliomas interaction of PKM2 with Cdk1 may play an important role in cell cycle progression. Finally, it will be important to validate the role of PKM2 in regulating Cdk1-cyclinB activation in other tumor types and to define in more detail how PKM2 binding contributes to Cdk1 activation, and perhaps more importantly, to determine if molecules can be developed that alter the interaction and can be used therapeutically to disrupt the elegant linkage between metabolism and cell cycle regulation necessary for tumor cell growth.

## Data Availability Statement

The original contributions presented in the study are included in the article/**Supplementary Material**. Further inquiries can be directed to the corresponding author.

## Author Contributions

Study conception and design: SO, YT, and JM. Acquisition of data: SO, TCJ, and YT. Analysis and interpretation of data: SO, TCJ, YT, and JM. Drafting of the manuscript: SO, YT, and JM. All authors contributed to the article and approved the submitted version.

## Funding

Psi Beta Psi Sorority to JM.

## Conflict of Interest

The authors declare that the research was conducted in the absence of any commercial or financial relationships that could be construed as a potential conflict of interest.

## Publisher’s Note

All claims expressed in this article are solely those of the authors and do not necessarily represent those of their affiliated organizations, or those of the publisher, the editors and the reviewers. Any product that may be evaluated in this article, or claim that may be made by its manufacturer, is not guaranteed or endorsed by the publisher.
