# Supplementary figures and images for: PKM2 Interacts With the Cdk1-CyclinB Complex to Facilitate Cell Cycle Progression in Gliomas
Source: Front Oncol. 2022 Mar 22;12:844861. doi: 10.3389/fonc.2022.844861 (PMC8981990; doi:10.3389/fonc.2022.844861)

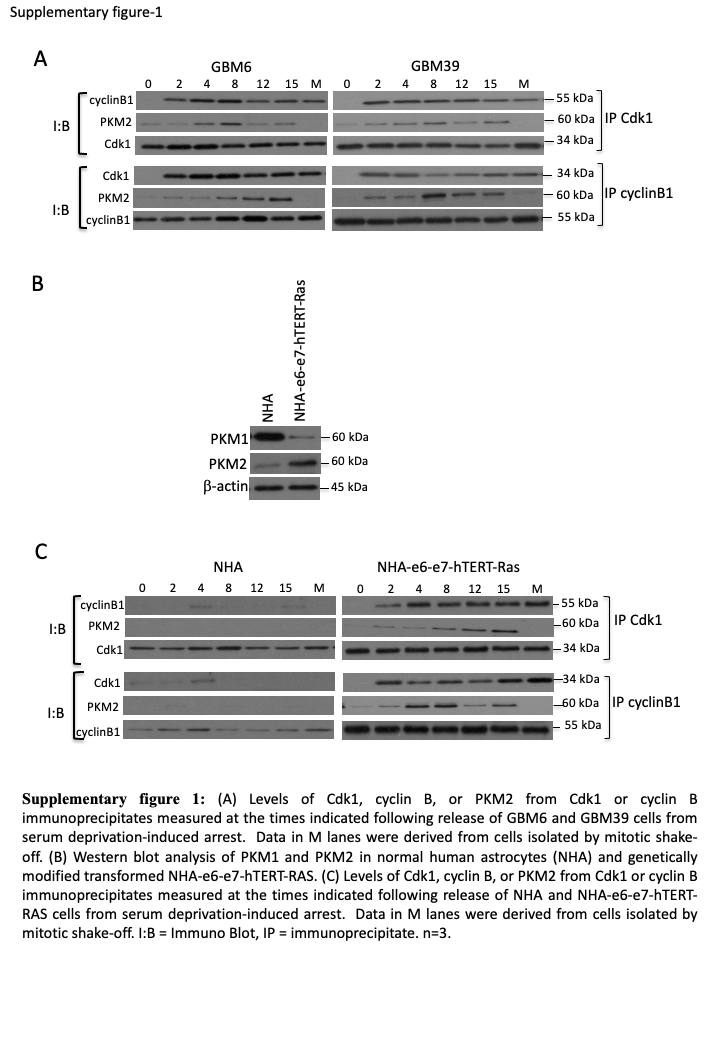

Supplement: Supplementary file 1 [file Image_1.tiff]
